# ASSOCIATIONS BETWEEN MEDIA CONSUMPTION AND AGEIST ATTITUDES: A CROSS-NATIONAL ANALYSIS

**DOI:** 10.1093/geroni/igac059.1603

**Published:** 2022-12-20

**Authors:** Erfei Zhao, Eileen Crimmins, Elizabeth Zelinski, Eunyoung Choi

**Affiliations:** University of Southern California, Leonard Davis School of Gerontology, Los Angeles, California, United States; University of Southern California, Los Angeles, California, United States; University of Southern California, Los Angeles, California, United States; School of Global Public Health, New York University, Los Angeles, California, United States

## Abstract

The mass media has been thought to be associated with public opinion, often creating and sustaining stereotypes. However, little is known about the role of media exposure in people’s ageist attitudes, particularly at a cross-national level. This study examines whether the daily use of different media types is associated with personal attitudes towards older people. We analyzed data from 59,103 adults across 54 countries, using the World Value Survey wave 6 (2010-2014). Personal ageist attitudes were assessed by whether participants agree that older people are a burden on society. We used logistic regression, controlling for individual- and country-level factors. Our findings suggest that people’s exposure to media is significantly associated with their attitudes towards older adults, but differently by the platform and respondents’ age. Those who used newspaper (OR:1.66, CI:1.38-1.98), magazines (OR:1.67, CI:1.27-2.20), and radio (OR:1.42, CI:1.23-1.65) were more likely to have negative attitudes toward older people, whereas those who used TV (OR:0.62, CI:0.53-0.72) and internet (OR:0.76, CI:0.65-0.89) were less likely to. Further, the effects of newspaper and radio consumption on people’s attitudes were moderated by respondents’ age. Younger adults’ ageist attitudes had a stronger negative relationship with these media types compared to those that are older. For the older age group, in contrast, more consumption of newspapers and radio are associated with less ageist attitudes. Future studies may focus on the content of each platform and assess their effect on people’s ageist attitudes by age groups in order to understand how to foster a more age-friendly media environment.

